# Mozart’s rhythm influence on Alzheimer’s disease progression via modulation of pathological damage and cognition

**DOI:** 10.1016/j.isci.2025.113168

**Published:** 2025-07-21

**Authors:** Junjun Li, Chuanjiang Wu, Qinjun Liu, Mingqi Liu, Ting Liu, Airui Li, Guangchao Fu, Zhiyong Zou, Daqing Guo, Ke Chen, Yang Xia, Dezhong Yao

**Affiliations:** 1 The Clinical Hospital of Chengdu Brain Science Institute, MOE Key Lab for Neuroinformation, School of Life Science and Technology, University of Electronic Science and Technology of China, Chengdu, China; 2 Department of Neurosurgery, Sichuan Provincial People’s Hospital, University of Electronic Science and Technology of China, Chengdu 611731, China; 3 School of Electrical Engineering, Zhengzhou University, Zhengzhou, China; 4 Research Unit of NeuroInformation, Chinese Academy of Medical Sciences, Chengdu, China

**Keywords:** Neuroscience, Behavioral neuroscience, Microbiome

## Abstract

Rhythm perception is considered a conserved trait across species, and musical rhythm exposure (MRE) has been demonstrated to enhance cognitive functions in healthy individuals. Alzheimer’s disease (AD), characterized by cognitive decline and pathological degeneration, may potentially be delayed by MRE. In this study, the APP/PS1 AD mouse model was exposed to Mozart’s K.448 rhythm for six months, with APP/PS1 and wild-type C57BL/6J mice serving as controls. The Morris water maze test was employed to assess the impact of MRE on spatial learning and memory. Pathological damage was evaluated through amyloid-beta and phosphorylated tau levels. Additionally, hippocampal microglia activation, inflammatory markers, and gut microbiota composition were analyzed. The study revealed that MRE improves cognitive function, reduces amyloid plaque accumulation, suppresses microglial activation and neuroinflammation, and modulates gut microbiota composition. This suggests that MRE offers a promising non-pharmacological approach to slowing cognitive decline and pathological damage in AD.

## Highlights

• Exposure to Mozart’s K.448 rhythm improves cognitive function in APP/PS1 mice
• Mozart’s K.448 rhythm reduces AD pathology and inflammation in APP/PS1 mice
• Mozart’s K.448 rhythm regulates gut microbiota in APP/PS1 mice

## Graphical abstract

**Figure:**
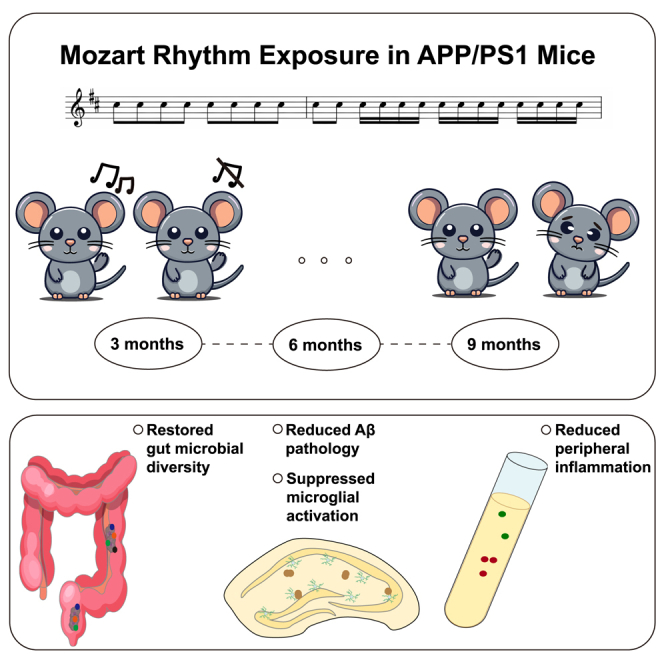

## Introduction

With global population aging, the incidence of Alzheimer’s disease (AD) is rising at an alarming rate.^1^ However, no effective therapeutic drugs are currently available to cure or halt the progression of AD. Considering the complexity of the etiology of AD and the extended developmental stage of the disease, nonpharmacological interventions could be used to prevent and alleviate the symptoms of AD.^2^^,^^3^^,^^4^ Music has been shown to influence biological systems, evoke pleasure, reduce anxiety, inspire movement, and foster social connections.^5^^,^^6^^,^^7^ Listening to music engages the whole brain through a diverse set of perceptive and cognitive operations and diverse neural substrates.^8^^,^^9^ Previous studies have demonstrated that music exposure serves as a therapeutic approach to treat psychiatric disorders, including depression,^10^^,^^11^ epilepsy,^12^^,^^13^^,^^14^ Parkinson’s disease,^15^^,^^16^^,^^17^ and senile dementia.^18^^,^^19^

For AD patients, music exposure can improve their emotional state,^20^ sleep quality,^21^ and cognitive performance.^22^ Exposure to Mozart K.448 reportedly improved performance on origami and paper-cutting tests in older patients with mild cognitive impairment.^23^ Moreover, musical intervention can enhance cognitive function and hippocampal levels of neurotrophic factors in rat models.^24^^,^^25^ However, one study found no significant difference between the effects of music and noise on oxidative stress and immune responses in animal models.^26^ These complex results may be due to the comprehensive features of music (rhythm, pitch, duration, timbre, etc.) and the extraction of key components from music as auditory intervention material is therefore of paramount importance. Among them, the significance of rhythm in eliciting behavioral responses to music has been substantiated.^24^

Among musical elements, rhythm, and temporal periodicity (sonic patterns that repeat regularly in time) are widely recognized across species. Human perception of musical rhythm involves detecting periodicity and producing precise temporal predictions of upcoming events.^27^ This ability to detect and predict auditory rhythms is central to music’s positive effect on a variety of neurologic disorders. Musical rhythm exposure (MRE) has been shown to normalize gait in Parkinson’s disease^28^^,^^29^ and to enhance language recovery after stroke^30^^,^^31^; rhythm-based musical training can improve executive function in preschoolers^32^ and improve language abilities in typically developing children and children with developmental dyslexia.^33^ However, the effects of musical rhythm on individuals with AD or mild cognitive impairment (MCI) remain largely unexplored. Two relevant studies showed that sensory stimuli with a frequency of 40 Hz can effectively improve clinical symptoms in patients with AD.^34^^,^^35^ As AD patients exhibit a range of physiological rhythm disorders,^33^^,^^36^^,^^37^ and music rhythm is a much more ecological stimulus than the monotonous 40 Hz rhythm, it may offer greater potential for delaying neurodegeneration. Interestingly, musical rhythms share structural similarities with key physiological rhythms.^38^^,^^39^^,^^40^ For example, human perception is known to be sensitive to 1/f structure in the environment.^40^^,^^41^ The works of classical composers exhibit a unique 1/f rhythm spectrum, such as Beethoven, Haydn, and Mozart.^42^ From a psychological point of view, composers have internalized some regularity of the physical world because of its ability to interact with biological systems.^43^ Thus, musical rhythm may influence the onset and progression of AD through this shared fundamental mechanism.

The gut microbiota demonstrates remarkable plasticity and sensitivity to changes in the external environment. It plays a critical role in regulating the gut-associated immune system, thereby influencing immune responses both systemically and within the central nervous system.^44^ Auditory stimuli can affect the composition of gut microbiota. Noise disrupts the homeostasis of the symbiotic microbiota and triggers oxidative inflammation.^45^ It is well established that peripheral inflammation can persistently activate microglia, impair neural function, and exacerbate the accumulation of β-amyloid protein (Aβ).^46^ Research has shown that music intervention can reduce the prevalence of pathogenic bacteria in the gut microbiota of mice and enhance the abundance of beneficial microbes.^47^ We further suppose the potential MRE effect may be attributed to its ability to sustain immune homeostasis related to the gut.

To validate these hypotheses, we employed APPswe/PSEN1dE9 (APP/PS1) mice as our experimental model, subjecting them to long-term MRE. The Morris water maze (MWM) was employed to assess spatial learning and memory performance. Furthermore, we examined the effects of MRE on AD progression by evaluating pathological markers, serum inflammatory cytokines, and changes in gut microbiota composition. This comprehensive approach allowed us to evaluate the potential therapeutic benefits of MRE in mitigating AD progression.

## Results

### MRE protected against cognitive function impairment in APP/PS1 mice

**Figure 1:**
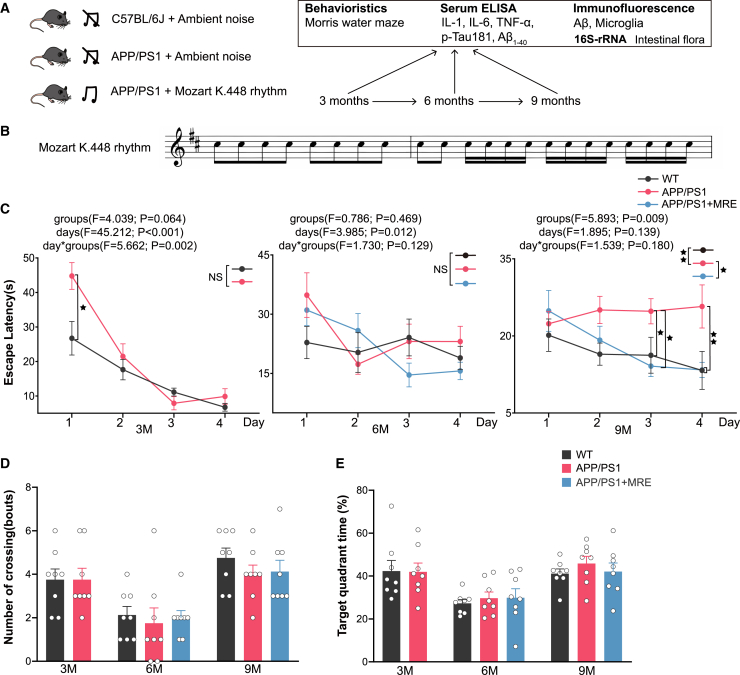
MRE protected against impaired cognitive function in APP/PS1 mice (A) Schematic diagram of the animal experiments. (B) Derived from the fifth and sixth stanzas of Mozart K.448, the rhythm adjusts the pitch of the piece to the same level as the original piece of music. (C) The average escape latency time was calculated as the amount of time needed to locate the submerged platform at 3, 6, and 9 months in mice in the MWM task. (D) The number of platform crossings in the MWM probe trials (no platform). (E) The time the mice spent in the target quadrant area in the MWM probe trials (no platform). *n* = 8. The data are reported as the means ± S.E.M. The data were analyzed by one-way ANOVA with the LSD multiple comparison test. ∗*p* < 0.05, ∗∗*p* < 0.01.

We utilized the MWM test to evaluate the effects of MRE on spatial learning and memory in APP/PS1 mice. At 3 and 6 months of age, there were no significant differences in escape latency among the three groups, indicating comparable learning abilities during these stages. By 9 months, the APP/PS1 group exhibited significantly impaired learning ability compared to the WT group (*p* < 0.01). At this time point, the APP/PS1+MRE group showed significantly improved learning ability compared to the APP/PS1 group (*p* < 0.05), with no significant difference observed between the APP/PS1+MRE and WT groups (Figure 1C). In the probe trial, no significant differences were observed among the groups in either the number of platform crossings or the time spent in the target quadrant at any of the assessed time points (Figures 1D and 1E).

### MRE effectively inhibited the rapid accumulation of Aβ and p-Tau181 in APP/PS1 mice

**Figure 2:**
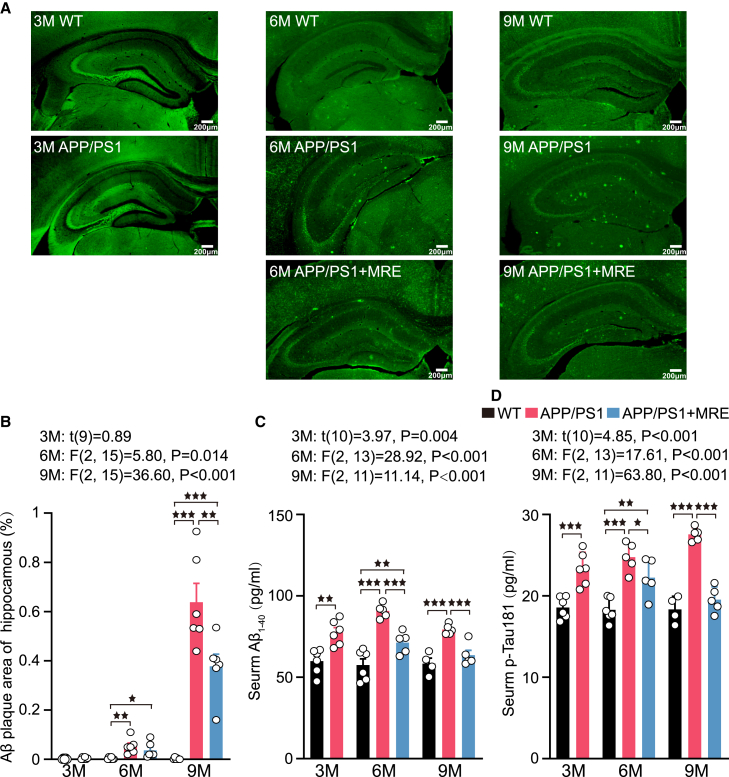
MRE effectively inhibited the rapid accumulation of Aβ and p-Tau181 in APP/PS1 mice (A) The area of Aβ was evaluated by immunofluorescence in hippocampal slices. Scale bar: 200 μm. (B) The area of Aβ within the hippocampus is a percentage of the entire hippocampal area in the slice (n = 5–6). For each mouse, 2–3 brain slices were analyzed, and the average value was used for quantification. (C and D) The levels of Aβ1−40 and p-Tau181 in the serum of each group of mice (n = 4–6) were determined by ELISA. One sample from the WT group at 9 months of age exhibited hemolysis and was therefore excluded from the analysis. The data are reported as the means ± S.E.M. The data were analyzed by one-way ANOVA with the LSD multiple comparison test. ∗*p* < 0.05, ∗∗*p* < 0.01, ∗∗∗*p* < 0.001.

At each time point, 5–6 mice per group were randomly selected for the assessment of AD pathology. At 3 months, APP/PS1 mice showed no significant Aβ plaque deposition in the hippocampus. Aβ deposition in the hippocampus was evident at 6 months (Figure 2A). At this stage, both the APP/PS1 group (*p* < 0.01) and the APP/PS1+MRE group (*p* < 0.05) exhibited significantly higher Aβ plaque levels compared to the WT group. At 9 months, the APP/PS1 group showed significantly larger Aβ plaque areas than the APP/PS1+MRE group (*p* < 0.01). Moreover, the Aβ plaque areas in the APP/PS1 group (*p* < 0.001) and APP/PS1+MRE group (*p* < 0.001, Figure 2B) were still greater than those in the WT group.

To further validate the effects of MRE on AD pathology, we measured serum levels of Aβ_1-40_ and p-Tau181 in mice. At 3 months, serum levels of Aβ_1-40_ and p-Tau181 were significantly elevated in APP/PS1 mice compared to WT mice (*p* < 0.01, *p* < 0.001). Following 3 months of MRE, serum Aβ_1-40_ level in the APP/PS1+MRE group were significantly reduced compared to those in the APP/PS1 group (*p* < 0.001). Similarly, serum p-Tau181 level were also significantly lower in the APP/PS1+MRE group than in the APP/PS1 group (*p* < 0.05). At 9 months, both Aβ_1-40_ and p-Tau181 levels remained significantly lower in the APP/PS1+MRE group than in the APP/PS1 group (*p* < 0.001 for both). There were no significant differences in Aβ_1–40_ and p-Tau181 levels between the APP/PS1+MRE group and the WT group (Figures 2C and 2D).

### MRE maintained the microglial population in the hippocampus of APP/PS1 mice at a homeostatic level

**Figure 3:**
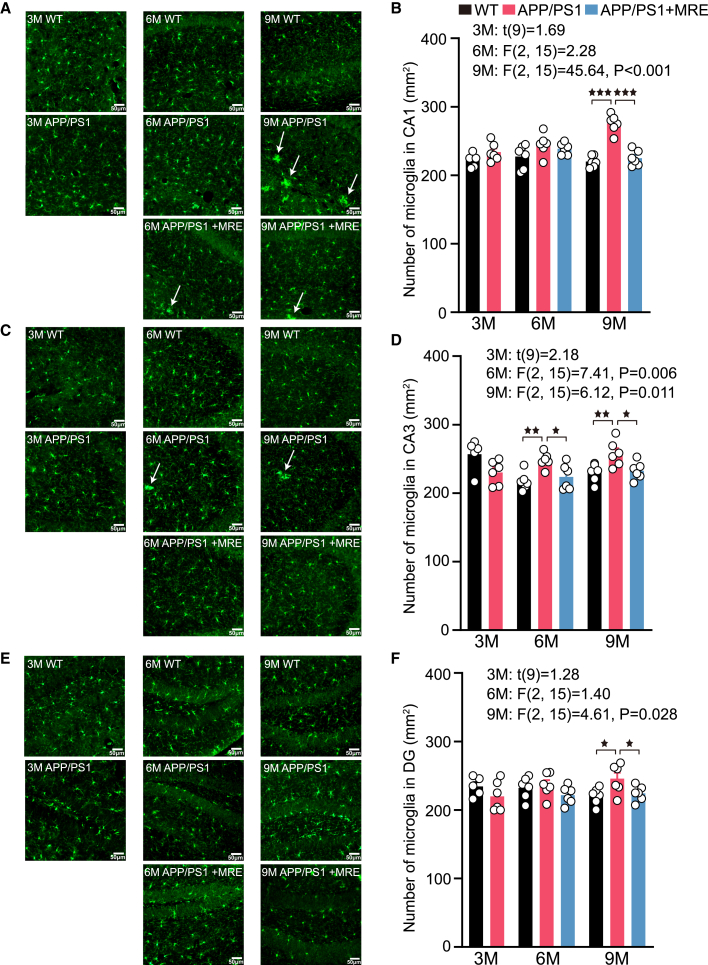
MRE maintained the microglial population in the hippocampus of APP/PS1 mice at a homeostatic level The number of microglia was evaluated by immunofluorescence in hippocampal CA1, CA3, and DG slices (A, C, and E), and the number of microglia in the corresponding regions is shown (B, D, and F). Scale bar: 50 μm. For each mouse, 2–3 brain slices were analyzed, and the average value was used for quantification. 5–6 mice per group. The data are reported as the means ± S.E.M. The data were analyzed by one-way ANOVA with the LSD multiple comparison test. ∗*p* < 0.05, ∗∗*p* < 0.01, ∗∗∗*p* < 0.001.

Microglial cells are considered the first line of defense in the brain’s immune system (Figures 3A–3C and 3E). At 3 months of age, no significant differences were observed in microglial counts in the CA1, CA3, and DG regions of the hippocampus between the APP/PS1 and WT groups. Statistical analysis indicated that abnormal microglial activation first emerged in the CA3 subregion in AD mice (Figure 3D). At 6 months, the number of microglia in the CA3 was significantly higher in the APP/PS1 group than in the WT group (*p* < 0.01). The APP/PS1+MRE group exhibited significantly fewer microglia in the CA3 subregion compared to the APP/PS1 group (*p* < 0.05). Moreover, we also found that the microglia exhibited aggregation (Figure 3C, arrow). At 9 months, the number of microglia in the CA1, CA3, and DG regions was significantly higher in the APP/PS1 group than in the WT group (*p* < 0.001, *p* < 0.01, *p* < 0.05, respectively). In contrast, the APP/PS1+MRE group showed significantly fewer microglia in these regions compared to the APP/PS1 group (*p* < 0.001, *p* < 0.05, *p* < 0.05; Figures 3B, 3D, and 3F).

### MRE effectively suppressed the rapid increase in peripheral inflammation in the APP/PS1 mice

**Figure 4:**
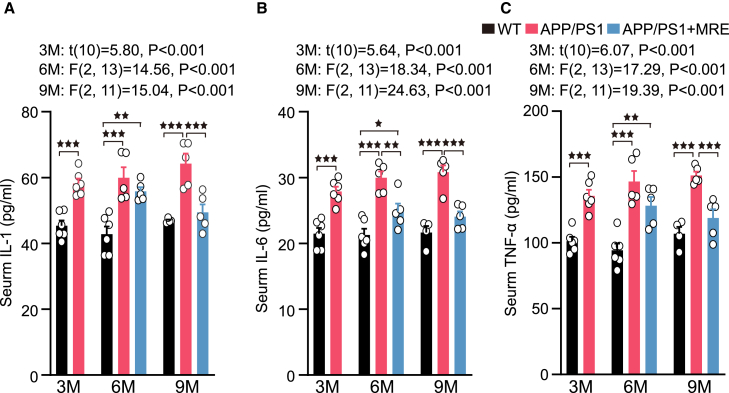
MRE effectively suppressed the rapid increase in peripheral inflammation in the APP/PS1 mice The expression levels of (A) IL-1, (B) IL-6, and (C) TNF-α in the serum of each group of mice (n = 4–6) were determined via ELISA. One sample from the WT group at 9 months of age exhibited hemolysis and was excluded from the analysis. The data are reported as the means ± S.E.M. The data were analyzed by one-way ANOVA with the LSD multiple comparison test. ∗*p* < 0.05, ∗∗*p* < 0.01, ∗∗∗*p* < 0.001.

The level of peripheral inflammation in the APP/PS1 mice significantly increased at 3 months, with the serum levels of IL-1, IL-6, and tumor necrosis factor-α (TNF-α) being significantly greater than those in the WT mice (*p* < 0.001 for all comparisons). At 6 months, both the APP/PS1 group (*p* < 0.001 for all comparisons) and the APP/PS1+MRE group (*p* < 0.01, *p* < 0.05, and *p* < 0.01, respectively) showed significantly elevated levels of IL-1, IL-6, and TNF-α relative to the WT group. Notably, the IL-6 level in the APP/PS1+MRE group was significantly reduced compared to that in the APP/PS1 group (*p* < 0.01). At 9 months, the serum levels of IL-1, IL-6, and TNF-α in the APP/PS1 group remained significantly higher than those in the WT group (*p* < 0.001 for all comparisons). The APP/PS1+MRE group exhibited significantly lower levels of IL-1, IL-6, and TNF-α compared to the APP/PS1 group (*p* < 0.001 for all comparisons), with no significant differences observed relative to the WT group (Figures 4A–4C).

### MRE enhanced the diversity and altered the composition of gut microbes in APP/PS1 mice

**Figure 5:**
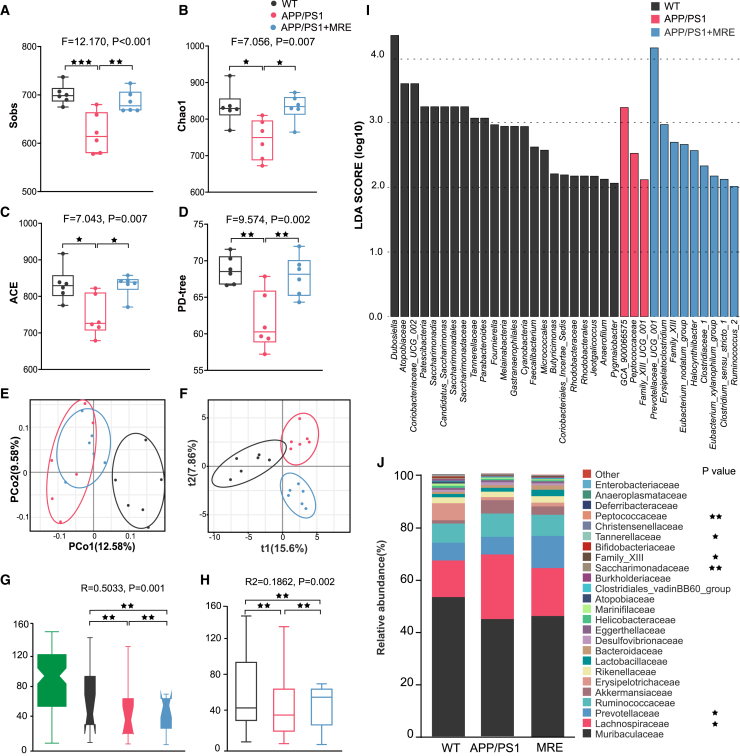
MRE enhanced the diversity and altered the composition of gut microbes in APP/PS1 mice (A–D) Sobs, Chao1, and Ace account for the species richness or the number of species presented, and the PD-tree reflects the diversity of sample lineages combined with evolutionary distance. (E) PCoA plot based on the unweighted UniFrac index at the OTU level. (F) PLS-DA plot based on the unweighted UniFrac index at the OTU level. (G) ANOSIM test based on the unweighted UniFrac index at the OTU level. (H) Adonis (PERMANOVA) test based on the unweighted UniFrac index at the OTU level. (I) The most differentially abundant taxa in each group were identified by LDA scores generated from the LEfSe analysis. (J) Relative abundances of predominant bacteria at the family level in each group. *n* = 6. Data are shown as box-and-whisker plots median and interquartile range. Kruskal-Wallis rank-sum test. ∗*p* < 0.05, ∗∗*p* < 0.01, ∗∗∗*p* < 0.001.

At 9 months, the richness of gut microbiota in each group were evaluated using the Sob, Chao1, ACE, and PD-tree indices. According to the Sob index, α-diversity in the APP/PS1 group was significantly lower than that in the WT group (*p* < 0.001) and the APP/PS1+MRE group (*p* < 0.01; Figure 5A). Based on the Chao1 diversity index, the APP/PS1 group was significantly lower than that in the WT (*p* < 0.05) and APP/PS1+MRE groups (*p* < 0.05; Figure 5B). On the basis of the ACE, the APP/PS1 group showed significantly lower α-diversity than the WT (*p* < 0.05) and APP/PS1 + MRE groups (*p* < 0.05; Figure 5C). The phylogenetic diversity (PD-tree) index further confirmed a significant reduction in microbial diversity in the APP/PS1 group compared to the WT and APP/PS1+MRE groups (*p* < 0.01 for both, Figure 5D). To assess β-diversity, we performed principal coordinate analysis (PCoA) and PLS-DA based on unweighted UniFrac distance matrices (Figures 5E and 5F). Based on Anosim and Adonis (PERMANOVA) tests, β diversity was assessed by grouping information, revealing significant differences in microflora structure and species composition between groups following long-term MRE exposure (Figures 5G and 5H). Linear discriminant analysis (LDA) was used to identify taxa that significantly differed among groups, revealing distinct microbial signatures at various taxonomic levels from domain to species (Figure 5I). At the family level, species with significantly different abundances across groups were visualized using stacked bar plots. The results showed that MRE can change the gut microbiome composition of AD mice, as illustrated in Figure 5J.

Additionally, at 3 months of age, significant differences in β-diversity were detected among the groups, indicating that early-stage AD mice already exhibited a distinct gut microbiota composition compared to controls (Figure S1). By 6 months, the APP/PS1 group exhibited significantly lower alpha diversity across multiple indices compared to the other two groups, with notable differences in beta diversity observed among all three groups (Figure S2). These findings further support that MRE effectively modulates gut microbiome homeostasis and prevents a decrease in microbial richness.

### MRE leading to the convergence of APP/PS1 mice toward WT mice

**Figure 6:**
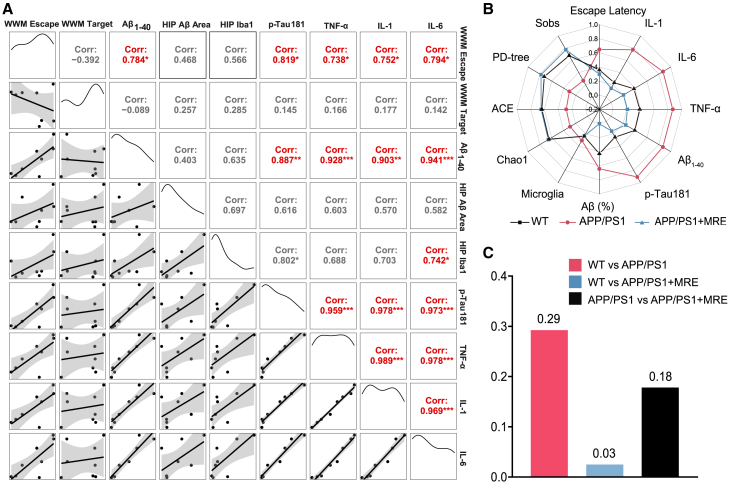
MRE leading to the convergence of APP/PS1 mice toward WT mice (A) Correlations among all the detection indices, according to the Spearman correlation analysis. (B) The normalized values of each detection index at age 9 months. (C) The mean squared error distance for the test indices for each group of mice aged 9 months ∗*p* < 0.05, ∗∗*p* < 0.01, ∗∗∗*p* < 0.001.

To determine the factors governing the improvement in learning ability in APP/PS1+MRE mice, we conducted a correlation analysis between escape latency and various pathological and inflammatory markers (Figure 6A). Our research corroborated the positive correlation between the escape latency of mice and pathological indices, including Aβ_1-40_ and p-Tau181 (r = 0.784, *p* < 0.05; r = 0.819, *p* < 0.05). The escape latency of the mice was significantly associated with inflammation levels, including TNF-α, IL-1, and IL-6 (r = 0.738, *p* < 0.05; r = 0.752, *p* < 0.05; and r = 0.794, *p* < 0.05, respectively). Furthermore, a significant correlation between the extent of pathological damage and the degree of inflammation was observed in the mice. In particular, significant correlations were detected between Aβ_1-40_ and TNF-α, IL-1, and IL-6 (r = 0.928, *p* < 0.001; r = 0.903, *p* < 0.01; and r = 0.941, *p* < 0.001, respectively). Similarly, significant correlations were detected between p-Tau181 and TNF-α, IL-1, and IL-6 (r = 0.959, *p* < 0.001; r = 0.978, *p* < 0.001; and r = 0.973, *p* < 0.001). These findings confirm that elevated inflammation impairs spatial learning and exacerbates pathological damage in AD.

Subsequently, we normalized the detection indices of the 9-month-old mice in each group and observed that the indices in the APP/PS1+MRE group exhibited greater resemblance to those in the WT group (Figure 6B). By calculating the mean squared error distance of the test indicators in mice aged 9 months, it was further demonstrated that long-term MRE caused the characteristics of the APP/PS1 mice to be more similar to those of the WT mice (Figure 6C).

## Discussion

### MRE and cognitive behavior

We observed that MRE delayed the onset of learning disabilities in AD mice. The escape latency serves as an indicator of the mouse’s learning ability and represents the encoding process of memory. Since the mice were trained to find the location of the underwater platform four times a day, this indicator could also reflect the level of short-term memory. Behavioral findings demonstrated that MRE had a positive impact on escape latency during the MWM test. It may be that rhythm regulates physiologic and behavioral functions through the mechanism of entrainment (synchronization of biological rhythms with musical rhythm based on acoustic resonance).^48^ Rhythm training can enhance neural activity associated with the encoding of short-term memory, engaging neural regions implicated in short-term memory processes in nonmusical tasks.^49^ In addition, the probe test revealed no statistically significant difference in long-term spatial memory capacity among the three cohorts of mice. We postulate that this observation may be attributed to the temporal developmental characteristics associated with cognitive impairment in the context of this disease. In our preliminary experiments, we found that 9-month-old APP/PS1 mice did not exhibit memory impairment in the new object recognition and social cognitive memory tests (Figure S3). Earlier studies suggested that APP/PS1 mice develop significant impairments in long-term memory at a later stage of the disease.^50^^,^^51^^,^^52^

### MRE and aging biomarkers

Long-term MRE had a positive effect on reducing the area of Aβ plaques in the hippocampus. At 9 months, the APP/PS1+MRE group had significantly fewer Aβ plaques in the hippocampus, which was consistent with the good escape latency of the MWM test. In multiple previous studies, mice with reduced pathological damage to the hippocampus performed better on cognitive behavioral tests.^53^^,^^54^^,^^55^

We observed early abnormal changes in the serum pathological level of AD mice, which occurred significantly earlier than they did in the brain and exhibited a declining trend under long-term MRE exposure. Correlation analysis revealed a close association between escape latency and serum pathological level in mice, suggesting that serum pathological alterations are promising predictors of cognitive ability. Aβ_1–40_ in the blood has been found to cooperate with neutrophils to trigger cognitive damage in AD.^56^ Blood p-Tau isoforms are reliable biomarkers for screening for AD in the absence of cognitive impairment.^57^^,^^58^ In summary, we hypothesize that pathological changes in AD first manifest in peripheral systems, subsequently progressing to the central nervous system, leading to brain pathology and cognitive decline. Long-term MRE may help alleviate AD-related pathology and promote a return to a normal physiological state.

### MRE and immune/inflammatory levels

Our experimental findings suggest that MRE could modulate immune-inflammatory responses. First, MRE significantly inhibited the abnormal proliferation of microglia in the hippocampus of AD mice. Second, it effectively reduced the level of inflammatory factors in the serum of AD mice. AD is also considered a chronic inflammatory disease, and the immune system plays a pivotal role in the onset and progression of this disease.^59^^,^^60^ In the C57 group, the number of microglia in the CA3 and DG hippocampal subregions was initially higher, with a subsequent decline over time. This trend is likely associated with neurodevelopmental dynamics. A study found a significant reduction in the number of newborn neurons in the DG between 3 and 6 months, along with a decrease in glial cells.^61^ The DG is crucial for adult neurogenesis, particularly during adolescence and early adulthood, when neural stem cell proliferation and neurogenesis are active.^62^ During this period, microglia play a key role in immune surveillance, synaptic pruning, and neural network remodeling.^63^ New neurons in the DG form highly plastic circuits by synaptically connecting with the CA3 region.^64^ As individuals mature, neurogenic activity diminishes, neural circuits stabilize, and microglial activity and numbers decrease.^65^ This process is closely linked to changes in microglial numbers during neurodevelopment. Thus, we hypothesize that similar changes in microglial numbers occur in both the DG and CA3 regions. In AD mice, the increasing number of microglia in the hippocampus is typically associated with the progression of the disease and the exacerbation of chronic inflammatory responses,^66^ potentially exhibiting a different pattern of glial cell quantity changes compared to wild-type mice. Proinflammatory molecules from the periphery may increase the brain’s inflammatory molecule pool through at least two mechanisms: via systemic circulation and/or neural pathways. If glial cells have been activated or initiated as seen in AD, stimulation by proinflammatory cytokines derived from the periphery will lead to exaggerated glial overexpression, accelerating the progression of AD.^67^

Intestinal mucosal lymphoid tissue is considered the largest and most important human immune organ. Specific bacterial strains can alter various inflammatory markers in the body,^68^ which affects brain development, function, and behavior during both health and disease.^69^^,^^70^ As anticipated, the positive intervention of musical rhythm significantly enhanced the abundance and richness of the gut microbiota in APP/PS1 mice, which indicated that the intestinal microbial composition of the APP/PS1+MRE group was changed. There are bidirectional connections between the central nervous system and the peripheral immune system.^71^ Interactions between the gut microbiota and host immune system in AD lead to the release of proinflammatory mediators, including cytokines and chemokines, as well as specific antibodies involved in regulating brain immunity. Alterations in the gut microbiota composition in APP/PS1 mice have been associated with elevated levels of Aβ in the central nervous system and impaired spatial learning and memory.^72^ In Tg2576 mice, dysregulation of the intestinal flora, dysfunction of the intestinal epithelial barrier, and deposition of Aβ in intestinal blood vessels were demonstrated to occur prior to the onset of cerebral Aβ deposition.^73^ This suggests that gut microbiota could serve as a sensitive indicator for assessing AD status. Moreover, MRE can effectively modulate immune homeostasis, attenuate inflammatory responses, and thereby help the mice resist central and peripheral immune dysfunction associated with AD.

### Rhythm and AD intervention

The distribution of electroencephalogram data in the frequency domain follows an inverse proportionality, and the slope of this linear relationship can serve as a measure of 1/f neural noise.^74^ The steepness (exponent) of 1/f activity has been linked to shifts in the balance between excitatory and inhibitory synaptic inputs in local neural circuits. In the temporal cortex, a sustained neural rhythm shows a 1/f structure, such that the logarithmic power decreases with increasing logarithmic frequency.^75^ In two studies, 1/f noise was interpreted as a reflection of youth and health, and departures from this power law are typically due to features of aging or chronic degenerative diseases that lead to increased health risks.^76^^,^^77^ For example, older adults exhibit a flatter and less pronounced negative 1/f slope compared to younger individuals.^78^ Regarding music, when the power spectrum of the musical note sequence exhibits an inverse relationship with frequency on a log-log plot, it is referred to as 1/f music. The power spectrum of loudness and frequency fluctuations in many classical music pieces follows a 1/f distribution across the average values of the entire composition.^79^ It was shown that the rhythmic components of music serve as crucial determinants of behavioral outcomes.^24^ Rhythm-based activities can be used to enhance general cognitive abilities in typically developing children.^80^ Studies with nonhuman primates and even zebrafish have shown that neural ensembles can entrain a rhythmic stimulus.^81^^,^^82^ For patients with AD, biorhythms and brain homeostasis shift to an imbalanced state characterized by the presence of Aβ plaques, activated microglia, altered network oscillations, and disrupted circadian rhythms.^83^^,^^84^^,^^85^ Music rhythm with a 1/f distribution tends to evoke more positive emotional responses in listeners compared to 1/f white noise or brown noise. Based on the correlation dimension and the largest Lyapunov exponent, research has shown that rhythmic variation has a greater impact on reducing the chaotic electrophysiological behavior in the brain, compared to melodic changes.^86^ The steepness of the 1/f activity increased following auditory stimulation,^78^ indicating that auditory material can serve as an intervention to influence brain activity. In addition, non-invasive 40 Hz frequency interventions can also compensate for the reduction in 40 Hz brain energy in AD mice through induction, thereby alleviating pathological and immune damage.^34^ As a spiritual product, the rhythm of music is more in line with the needs of biology. The organization of neural rhythms bears a striking resemblance to the organization of musical rhythms.^87^^,^^88^^,^^89^ Long-term MRE may follow this pattern, amplifying the effective components of the music and more effectively slowing the progression of cognitive decline in AD mice.

In summary, our findings demonstrate that APP/PS1 mice exhibit progressive deterioration in key indices with aging. MRE treatment effectively mitigates spatial learning deficits, immune dysfunction, and pathological progression in AD mice, resulting in phenotypic characteristics more akin to wild-type mice. These results highlight MRE as a promising noninvasive therapeutic strategy for AD, offering an ecologically sustainable, cost-effective, and scalable approach, particularly beneficial for high-risk individuals. When combined with pharmacological treatments, MRE represents a viable early intervention for those predisposed to AD.

### Limitations of the study

Future investigations should employ diverse behavioral paradigms to comprehensively assess MRE’s impact on AD-related cognitive decline, with particular attention to its effects on memory in advanced AD stages. Additionally, various musical materials will be incorporated to evaluate the universality of rhythm-based interventions and to select the optimal rhythmic elements for improving treatment outcomes.

## Resource availability

### Lead contact

Further information and requests for resources and reagents should be directed to and will be fulfilled by the lead contact, professor Dezhong Yao (dyao@uestc.edu.cn).

### Materials availability

This study did not generate unique reagents.

### Data and code availability

• 16S rRNA gene sequencing data have been deposited at the NCBI sequence read archive and are publicly available as of the date of publication. The accession number is PRJNA1254874 and is listed in the key resources table.
• All original code is available in this paper’s supplemental information.
• Any additional information required to reanalyze the data reported in this paper is available from the lead contact upon request.

## Acknowledgments

This research was supported by STI 2030-Major Projects 2022ZD0208500, the 10.13039/501100001809National Natural Science Foundation of China (no. 82072011), 10.13039/501100001809NSFC U24A20274, and the Sichuan Science and Technology Program (2024YFHZ0359).

## Author contributions

Study conception and/or design of the work: J.L., K.C., Y.X., and D.Y.; data acquisition, analysis and/or interpretation of data: J.L., C.W., and T.L.; drafting and/or revising the manuscript: J.L., K.C., Y.X., and D.Y.; all co-authors have read and approved the final version of the manuscript.

## Declaration of interests

The authors declare no competing interests.

## STAR★Methods

### Key resources table

**Table:** 

REAGENT or RESOURCE	SOURCE	IDENTIFIER
**Antibodies**		

Rabbit monoclonal anti-Iba-1	Abcam	Cat#ab178847; RRID: AB_2636859
Rabbit monoclonal anti-β-Amyloid	Cell Signaling Technology	Cat#8243S; RRID: AB_2797642
Goat Anti-Rabbit IgG (H + L) Antibody	ThermoFisher	Cat#A-11008; RRID: AB_143165

**Chemicals, peptides, and recombinant proteins**		

Mounting Medium, antifading (with DAPI)	Solarbio	Cat#S2110

**Critical commercial assays**		

Mouse IL-1 ELISA Kit	Abcam	Cat#ab197742
Mouse IL-6 ELISA Kit	Abcam	Cat#ab222503
Mouse p-TAU181 ELISA KIT	Shanghai Enzyme-linked Biotechnology Co., Ltd	Cat#ml106037
Mouse Aβ1-40 ELISA KIT	Shanghai Enzyme-linked Biotechnology Co., Ltd	Cat#ml001859
Mouse TNF alpha ELISA Kit	Abcam	Cat#ab208348

**Deposited data**		

16S rRNA gene sequencing of gut microbiota	This paper	NCBI SRA: PRJNA1254874; https://www.ncbi.nlm.nih.gov/bioproject/PRJNA1254874/

**Experimental models: Organisms/strains**		

Mouse: APPswe/PSEN1dE9 mice	Beijing HFK Bioscience Co., Ltd.	APP/PS1 transgenic mice (C57BL/6 background)
Mouse: C57BL/6J mice	Beijing HFK Bioscience Co., Ltd.	C57BL/6 colony

**Software and algorithms**		

SPSS 25.0	IBM SPSS	N/A
ImageJ	National Institutes of Health	https://imagej.net/ij/
Smart 3.0 tracking system	Panlab	N/A
Python 3.12	Python.org	https://www.python.org

### Experimental model and study participant details

#### Animals

Three-month-old male APP/PS1 mice and C57BL/6J mice were purchased from Beijing HFK Bioscience Co., Ltd. All mice were housed at 22 ± 1°C under a 12-h light/dark cycle with free access to food and water. All experiments were conducted following international standards on animal welfare and the guidelines for Animal Experiments of the Ethics Committee of Animal Experiments of the University of Electronic Science and Technology of China (1061 423 030 125 881).

In all experiments, “n” refers to the number of mice unless stated otherwise. In this study, C57BL/6J mice and APP/PS1 mice were first subjected to the MWM (*n* = 8), followed by the measurement of hippocampal Aβ plaques (n = 5–6), serum Aβ_1-40_, phosphorylated tau181 (p-Tau181), interleukin-1 (IL-1), interleukin-6 (IL-6), and tumor necrosis factor-α (TNF-α) (n = 4–6). Then, according to their genotypes, the remaining three-month-old mice were randomly divided into the following three groups: wild type (C57BL/6J) with natural noise (WT), APP/PS1 with natural noise (APP/PS1), and APP/PS1 with Mozart rhythm exposure (APP/PS1+MRE). And began to receive musical rhythm intervention. At 6 and 9 months, the same assessments were repeated. Additionally, fecal samples were collected from each group at each time point for gut microbiota analysis (n = 5–6). The experimental procedures are listed in Figure 1A.

### Method details

#### Auditory stimulation and treatments

The experimental study used the rhythm of Mozart’s K.448 (Figure 1B), which was taken from Mozart’s piano sonata K.448, preserving only the rhythm component. The musical materials were created by professionals from a conservatory. In this study, both the original and rhythm-only versions of the music were produced using the digital audio workstation Cubase (Steinberg Media Technologies GmbH, DE). The rhythmic components were extracted from Mozart’s composition K.448. To standardize pitch across all tones, the musical notes were transposed to “Middle C,” while preserving the original rhythm, tempo, dynamics, and timbre. The MIDI files were rendered into high-quality audio using the Galaxy Steinway VST (Galaxy Instruments, DE), with all default parameters retained and no additional processing (e.g., equalizer or modulation effects) applied. The final audio was exported from the MIDI project.

The APP/PS1 + MRE group was exposed to the Mozart K.448 rhythm from 20:00 to 00:00 every night for a total of 6 months. This intervention schedule was designed to align with the mice’s circadian rhythm, matching their natural peak activity during the dark phase.^90^ This is aimed at enhancing their perception of rhythmic auditory stimuli and the responsiveness of their nervous system, thereby enabling a more effective assessment of the potential impact of MRE on neural function in mice. The intensity of the musical rhythm stimulation was 65–75 dB, and the ambient noise level was approximately 65 dB. The waveforms and spectrograms were generated using Adobe Audition (Adobe Inc., USA), while power spectral density analysis was performed using Python software (Figure S4). The code for the power spectral density analysis is provided in the supplementary materials (Data S1).

#### Morris water maze

Learning and memory ability were assessed via the MWM test. The water temperature was consistently maintained at 22 ± 1°C. Mice underwent 16 training trials over 4 days, with four sessions daily, to locate a hidden platform. They were gently introduced into the water facing the wall from one of four quadrants. Successful platform discovery within 60 s allowed a 5-s stay; otherwise, mice were placed on the platform for 20 s. A 1-min probe trial was conducted 24 h post-training, with the platform removed to assess free swimming behavior. Mouse movements were recorded via video and analyzed using the Smart 3.0 tracking system (Panlab, ES). Learning efficacy was measured by escape latency, where shorter latencies indicated better performance. Memory was evaluated during the probe test by time spent in the target quadrant and platform location crossings.

#### Sample collection

Mice were anesthetized via intraperitoneal injection of pentobarbital sodium. Blood was collected through eyeball extraction using tweezers, followed by serum isolation via centrifugation. Subsequently, mice were perfused with 4% paraformaldehyde, and brain tissues were dissected and dehydrated using a sucrose gradient. The hippocampus was sectioned into 25 μm-thick slices and stored in phosphate-buffered saline at 4°C. Fecal samples were collected and stored at −80°C. Gut microbiota composition was analyzed using 16S rRNA gene sequencing.

#### Enzyme-linked immunosorbent assay (ELISA)

The concentrations of IL-1, IL-6, TNF-α, p-Tau181, and Aβ_1−40_ in the serum were determined using ELISA kits following the manufacturer’s guidelines. Serum (50 μL) was added to 96-well plates, along with 100 μL of horseradish peroxidase (HRP)-labeled detection antibody in both standard and sample wells. The plates were sealed, incubated at 37°C for 60 min in a water bath, and subsequently terminated with tetramethylbenzidine and stop solution. Between steps, the wells were washed. The absorbance (OD) was measured at 450 nm using a microplate reader, and sample concentrations were calculated. The minimal concentrations of IL-6 and p-Tau181 were less than 0.1 pg/mL, while those of IL-1, TNF-α, and Aβ1−40 were less than 1 pg/mL.

#### Immunofluorescence (IF) staining

Frozen sections were stained for Iba-1 and Aβ using IF. For IF staining, sections were immersed in 3% BSA (Thermo Fisher Scientific, MA) for 60 min at room temperature to block nonspecific antigens and then incubated with anti-Iba-1 monoclonal antibody (rabbit, 1:100, ab178847, Abcam) or anti-beta amyloid antibody (rabbit, 1:1000, 8243S, Cell Signaling Technology) overnight at 4°C. After the sections were rinsed three times in PBS, they were incubated with fluorochrome-conjugated secondary antibodies (1:500) (anti-rabbit, Alexa Fluor 488 for Iba-1 and Aβ; Thermo Fisher, USA) in a cassette at room temperature for 1 h. Then, the slices were covered with an anti-fluorescence attenuator containing DAPI (Solarbio, CHN). Finally, Iba-1 and Aβ signals were examined via fluorescence microscopy (OLYMPUS, JPN). The Aβ plaque area percentage was calculated by outlining the entire hippocampus in the images using ImageJ software and determining the proportion of the Aβ plaque area relative to the total hippocampal area. For the microglia cell count, 2–3 brain slices from each mouse were selected. In each slice, three random 100 × 100 μm regions were marked in the hippocampal CA1, CA3, and DG subregions. The number of microglia cells within each region was counted, and the average value was calculated. The results were then converted to a 1 mm^2^ unit for comparative analysis.

#### 16S rRNA high-throughput sequencing

Fecal samples from each group of mice were collected for sequencing. Bacterial DNA extraction, PCR amplicon sequencing, and genetic analysis were carried out by the Genedenovo Biotechnology Company (Guangzhou, China). The V3 + V4 domains of 16S rRNA were amplified by the barcoded specific primers 341F (CCTACGGGNGGCWGCAG) and 806R (GGACTACHVGGGTATTCTAAT). The intestinal microbiota was analyzed on the OmicSmart metagenome analysis platform (http://www.omicsmart.com). After obtaining the raw reads by sequencing, the low-quality reads were filtered, and the remaining sequences were assembled and filtered to ensure that the most effective data were utilized when clustering the reads into operational taxonomic units (OTUs). The clean tags were clustered into OTUs of ≥97% similarity. The OTUs were subsequently annotated via α-diversity analysis, β-diversity analysis (principal coordinate analysis (PCoA) and partial least squares discrimination analysis (PLS-DA), and the analysis of differences between groups was performed. The raw sequencing data have been submitted to NCBI Sequence Read Archive (SRA: PRJNA1254874).

### Quantification and statistical analysis

The data in the figures are expressed as the mean ± S.E.M. (standard error of the mean) and were analyzed using SPSS 25.0 (IBM, USA). Comparisons between two groups were performed by Student’s t test, and comparisons of more than two groups were performed by repeated measures one-way analysis of variance. Moreover, a post hoc least significant difference (LSD) test was used to determine statistically significant differences.

The Kruskal‒Wallis rank-sum test was used for α diversity analysis, and Tukey’s honest significant difference (HSD) test was used for pairwise posterior tests after multigroup comparisons. The relative abundances of gut microbiota at the family level were compared among groups using the Kruskal–Wallis test. A *p* value <0.05 was the threshold for statistical significance.

## Supplemental information

Document S1. Figures S1–S4

Data S1. Python script for power spectral density analysis of auditory stimuli
