# Supplementary material for: Mozart’s rhythm influence on Alzheimer’s disease progression via modulation of pathological damage and cognition
Source: iScience. 2025 Jul 21;28(8):113168. doi: 10.1016/j.isci.2025.113168 (PMC12335960; doi:10.1016/j.isci.2025.113168)
Supplement: Document S1. Figures S1–S4 [file mmc1.pdf]

iScience, Volume *28*

Supplemental information

Mozart's rhythm influence on Alzheimer's disease progression via modulation of pathological damage and cognition

Junjun Li, Chuanjiang Wu, Qinjun Liu, Mingqi Liu, Ting Liu, Airui Li, Guangchao Fu, Zhiyong Zou, Daqing Guo, Ke Chen, Yang Xia, and Dezhong Yao

Supplemental Figure

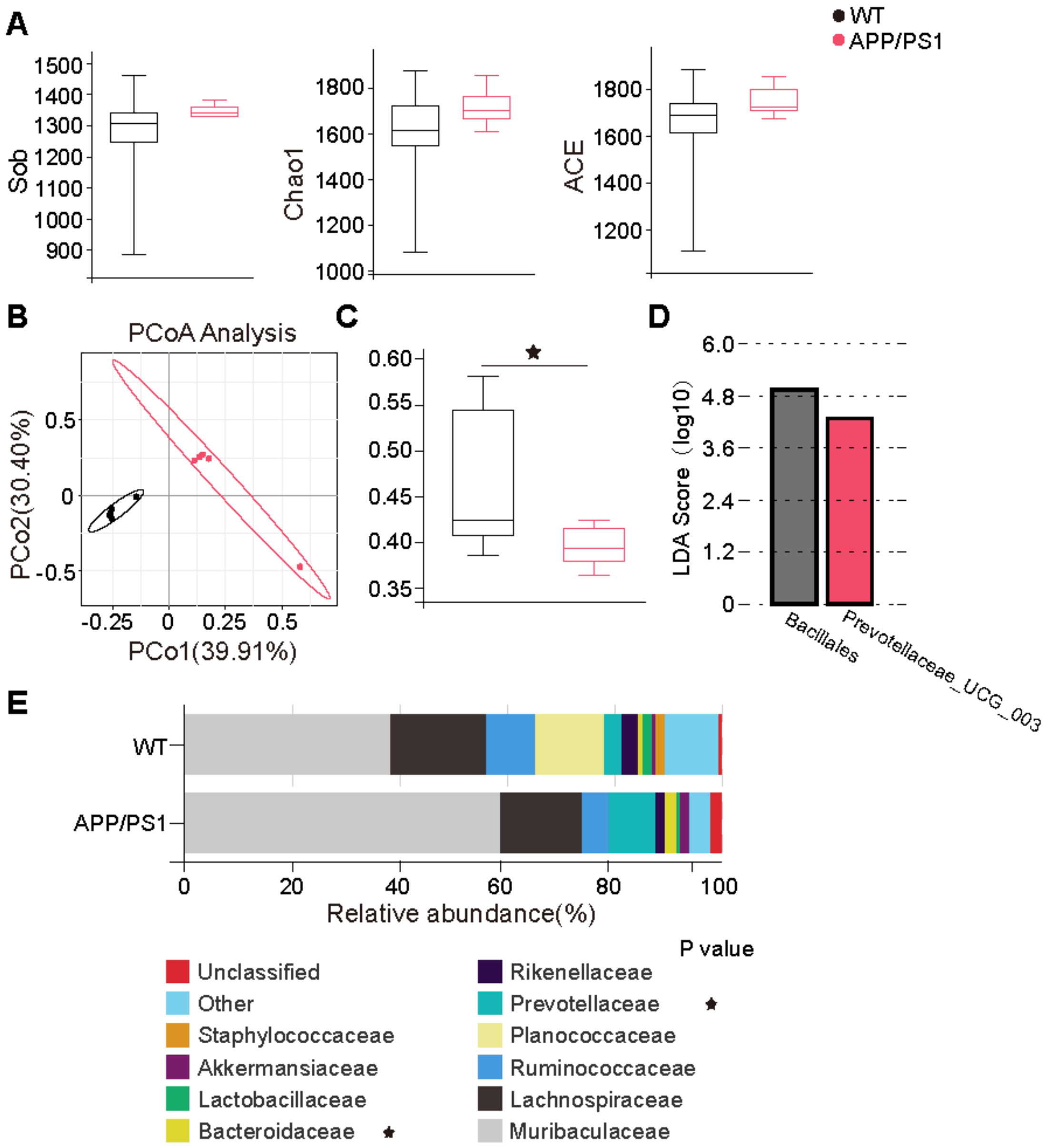

Fig. S1. Gut microbiome analysis at 3 months of age. (A) α-diversity analysis based on Sob, Chao1, and ACE indices. (B) PCoA plot based on the unweighted UniFrac index at the OTU level. (C) Adonis (PERMANOVA) test based on the unweighted UniFrac index at the OTU level. (D) The most differentially abundant taxa in the two groups were identified by LDA scores generated from the LEfSe analysis. (E) Relative abundances of predominant bacteria at the family level in each group. Wilcoxon rank-sum test. n=5. *P<0.05.

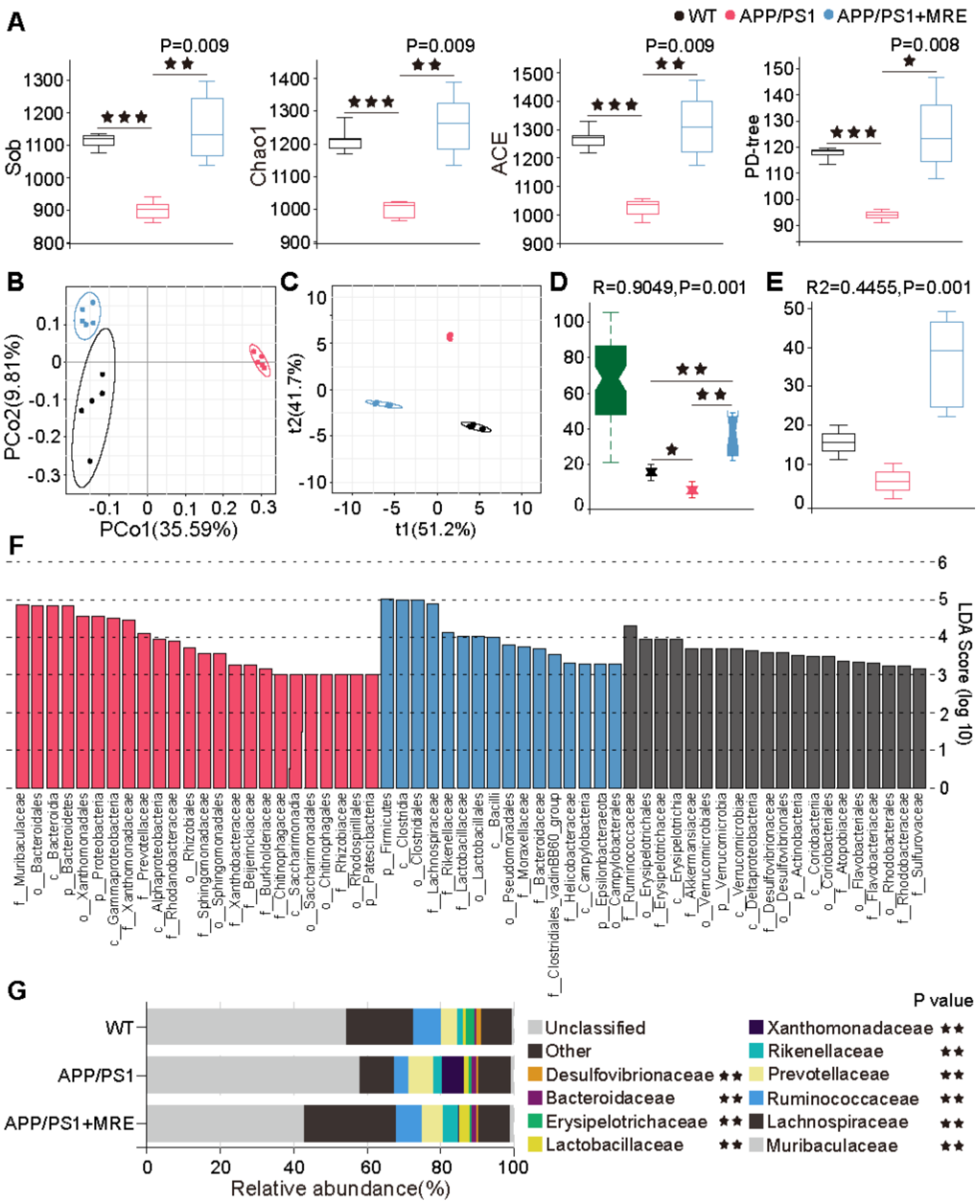

Fig. S2. Gut microbiome analysis at 6 months of age. (A) α-diversity analysis based on Sob, Chao1, ACE and PD-tree indices. (B) PCoA plot based on the unweighted UniFrac index at the OTU level. (C) PLS-DA plot based on the unweighted UniFrac index at the OTU level. (D) ANOSIM test based on the unweighted UniFrac index at the OTU level. (E) Adonis (PERMANOVA) test based on the unweighted UniFrac index at the OTU level. (F) The most differentially abundant taxa in each group were identified by LDA scores generated from the LEfSe analysis. (G) Relative abundances of predominant bacteria at the family level in each group. n=5. Kruskal-Wallis rank sum test. **P*<0.05, ***P*<0.01, ****P*<0.001.

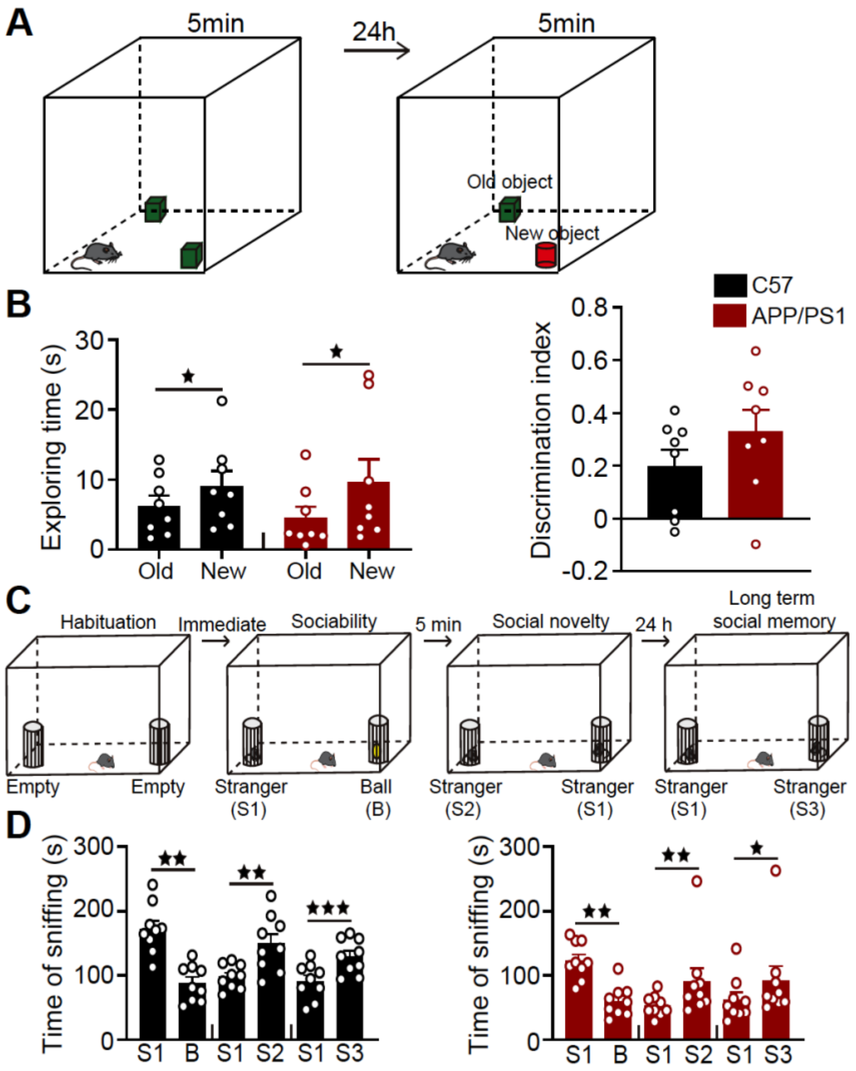

Fig. S3. Cognitive behavior testing in 9-month-old mice. (A) Schematic of the new object recognition task. (B) Exploration time of new and old objects, paired t-test; discrimination index, unpaired t-test. n=8. (C) Schematic of the social cognitive behavior task. (D) Exploration time during the social cognitive behavior task, including social, social novelty, and long-term social memory phases. Paired t-test. n = 9. The data are reported as the means ±S.E.M. **P*<0.05, ***P*<0.01, ****P*<0.001.

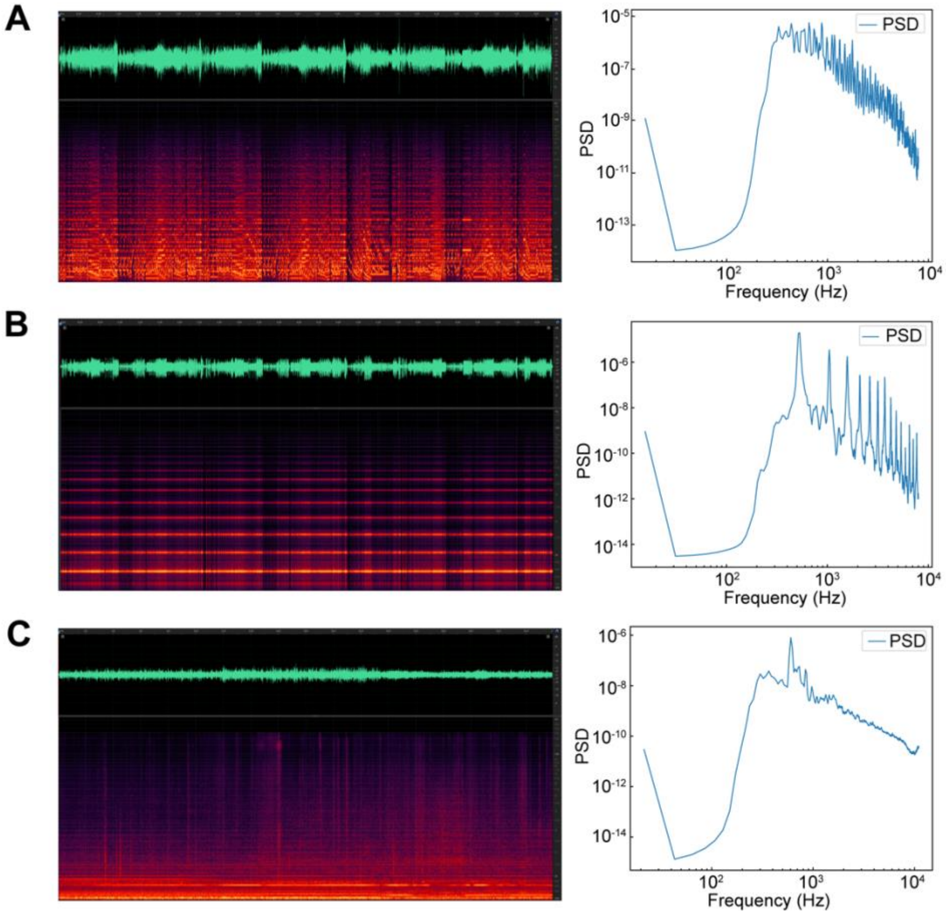

Fig.S4. Waveform, spectrogram, and power spectral density of the auditory materials. (A) Mozart K.448. (B) Mozart K.448 rhythm. (C) Natural noise.
